# Has a river dam affected the life‐history traits of a freshwater prawn?

**DOI:** 10.1002/ece3.6388

**Published:** 2020-06-20

**Authors:** Gicelle M. F. Silva, Marcelo C. Andrade, Breno R. M. Silva, Ingrid S. Palheta, Liziane B. Gonçalves, Rossineide M. Rocha, Maria A. P. Ferreira

**Affiliations:** ^1^ Laboratório de Imunohistoquímica e Biologia do Desenvolvimento Universidade Federal do Pará Belém Brazil; ^2^ Laboratório de Ultra Estrutura Celular Universidade Federal do Pará Belém Brazil; ^3^ Laboratório de Biologia Pesqueira e Manejo dos Recursos Aquáticos Grupo de Ecologia Aquática Belém Brazil

**Keywords:** abiotic factors, dammed river, estuary, freshwater prawn

## Abstract

In recent years, species richness and diversity in aquatic ecosystems has declined as environments are increasingly impacted by anthropic actions. Freshwater prawns are well adapted to survive in a disturbed and heterogeneous environment. For instance, Amazon river prawn (*Macrobrachium amazonicum*) populations vary in migratory behavior between rivers and estuaries, depending on factors such as dams. However, there is limited information on the influence of environmental conditions on life‐history traits of this species, which we investigate here using two distinct and unconnected aquatic systems, a dammed river and an estuary, in the eastern Brazilian Amazon. The biological characteristics of *M. amazonicum* populations in the two environments were compared and related to environmental parameters, which differed significant differences between the two environments and between seasons. Dissolved oxygen, precipitation, and temperature varied most significantly with the seasons in both the estuary and river. *M. amazonicum* prawns in the estuary were larger and heavier than those in the river during rainy periods. The mass–length ratios and condition factor varied significantly between the *M. amazonicum* populations in the estuary and river, with negative allometric growth (grows faster in length than in weight) predominating in both populations, and condition factor was better in the estuary for males and in the river for females. The relative frequencies of occurrence of the different female maturation stages and the male morphotypes were related to precipitation and turbidity in both environments and also to salinity in the estuary. In these two distinct aquatic systems, the abiotic parameters determined by the seasonal precipitation cycle profoundly influenced the development of this crustacean, despite its ecological plasticity. Overall, the study showed that river damming triggered environmental changes in the freshwater river ecosystem and played a key role in determining the life‐history characteristics of *M. amazonicum* in these contrasting aquatic systems.

## Graphical abstract

*Macrobrachium amazonicum* is endemic of South America with a large geographical distribution and both biological and economical importance to Amazon. It shows variations in the ecological characteristics, behavior, and life‐history traits between populations.

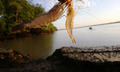

## 1 INTRODUCTION

In recent years, species richness and diversity in freshwater ecosystems has declined as the anthropic impact on these environments has grown (Dudgeon et al., 2006; Hughes, 2015; Pringle, 2001). One prominent example of this type of impact is river impoundments that transform lotic systems to lentic environments, forming very deep artificial habitats that generally support an increase in primary productivity, proliferation of floating macrophytes, high availability of food resources, and abundant aquatic fauna (Agostinho, Gomes, Santos, Ortega, & Pelicice, 2016; Agostinho et al., 1999; Kubečka, 1993). These environments are characterized by thermal stratification, lower dissolved‐oxygen concentrations in the deeper strata, dispersal of species (Agostinho, Pelicice, & Gomes, 2008; Wang, Duan, Liu, Chen, & Liu, 2013), and major alterations in water quality (Cunha‐Santino, Bitar, & Bianchini, 2013; Manyari & Carvalho, 2007; Wohl, Lane, & Wilcox, 2015). In contrast, estuaries are more natural environments that are rich in sediments, nutrients, and organic material and have a marked salinity gradient (Boto & Wellington, 1984; McKenney, 1996), which creates an enormous diversity of resources that supports the reproduction and growth of aquatic species (Telesh & Khlebovich, 2010).

In both types of aquatic systems, estuary and river, the environment may affect the life cycles of species, although few data are available on the effect of specific environmental factors in limiting the potential for survival or adaptation of local species. Niche theory posits that environmental conditions and local variations associated with the intrinsic characteristics of a species will determine its adaptation to the environment. These conditions are related to the microhabitats, abiotic factors, resources, and predators, all of which may be essential for the physiological and behavioral adaptation of species (Grinnell, 1917). Based on this theory, the species composition of local assemblages would be determined by the environmental factors that filter out the species incapable of establishing local populations (Hutchinson, 1957).

For aquatic invertebrates, abiotic variables of the water, in particular salinity, pH, temperature, and dissolved oxygen, affect the osmotic balance of the organism and its absorption of nutrients and may cause stress (Bernard, 1983; Cheng, Yeh, Wang, & Chen, 2002; Yang, Sierp, Abbott, Li, & Qin, 2016). In tropical estuaries and coastal regions where temperatures are constant, salinity is one of the principal factors determining growth (Oliveira et al., 2018). In dammed rivers, salinity is zero and all trophic levels are affected by a sharp drop in nutrient availability (Agostinho et al., 1999; Cunha‐Santino et al., 2013). Turbidity is related to suspended‐material concentrations, which influence the ecology of the aquatic environment (Göransson, Larson, & Bendz, 2013). All these parameters are affected by variations in rainfall, flow rate, vegetation cover, and soil (Hamilton & Luffman, 2009; Silva, Angelis, Machado, & Waichaman, 2008). Therefore, the physical and chemical characteristics of the environment may be important in determining the biometric characteristics of local aquatic animal species and their strategies for maintenance, growth, and reproduction (Deekae & Abowei, 2010; Fearnside, 2015; Kubečka, 1993).

Freshwater prawns are among the aquatic organisms most capable of adapting their morphophysiological and behavioral characteristics for survival in a heterogeneous environment. Members of the genus *Macrobrachium* generally exhibit amphidromy, in which females migrate to the estuary, where prolonged larval development occurs, followed by migration of postlarvae upstream to freshwater habitats. However, some *Macrobrachium* populations in rivers do not show amphidromy, due to factors such as the distance from an estuary or dam, and have low fecundity with shortened larval stages, completing their entire life cycle in fresh water (Anger, 2003; Murphy & Austin, 2005; Vergamini, Pileggi, & Manelatto, 2011). *M. amazonicum* shows high ecological and morphological plasticity (Freire, Bentes, Fontes, & Martins, 2017; Soeiro, Rocha, Maciel, Abrunhosa, & Maciel, 2016; Vergamini et al., 2011). This species occurs in the turbid water of large rivers such as the white‐water rivers in the Amazon basin (Collart & Magalhães, 1994; Magalhaes, 1985). The fecundity of this prawn has been reported to be lower in dammed rivers (Silva, Cintra, & Muniz, 2005; Silva, Jacobucci, & Mossolin, 2017) than in other rivers (Meireles, Valenti, & Mantelatto, 2013) and estuaries (Da Silva, Sampaio, & Santos, 2004; Lucena‐Frédou, Rosa Filho, Silva, & Azevedo, 2010). However, information about the influence of abiotic factors on *M. amazonicum* in different environments is still sparse.

**FIGURE 1:**
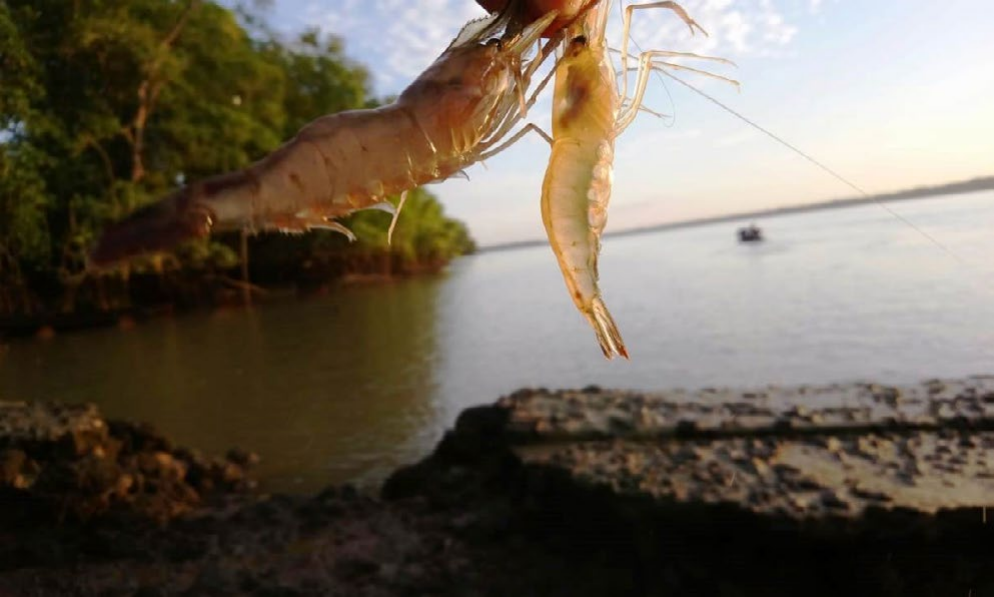
Photographs of the freshwater prawn *Macrobrachium amazonicum* in the estuary

**FIGURE 2:**
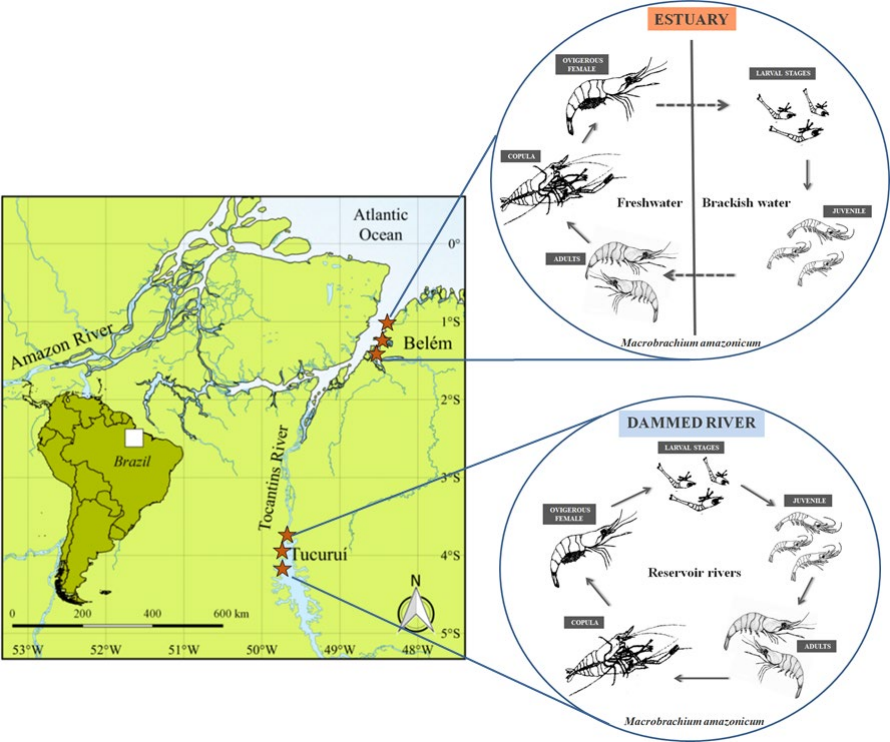
Location of sampling points and type of life cycle of *Macrobrachium amazonicum* at the two study sites in the state of Pará, northern Brazil. Estuary: The Furo das Marinhas estuary in Belém. River II: Tocantins River upstream from the Tucuruí hydroelectric dam

*Macrobrachium amazonicum* (Figure 1) shows differences in size and life cycle (Figure 2) when living in each environment separately (Bernard, 1983; Göransson et al., 2013). Data about the effect of the environment on life‐history traits of the prawn are important for understanding the survival dynamics of the species in different aquatic systems. Based on these considerations, we tested the hypothesis that river damming changes the environmental characteristics of aquatic systems and the life cycles of crustacean populations. To test this hypothesis, the present study investigated the influence of environmental conditions in two distinct and unconnected aquatic systems (river and estuary), with different salinity, during a seasonal cycle, on local populations of *M. amazonicum* in eastern Brazilian Amazonia.

## 2 MATERIAL AND METHODS

### 2.1 Study area

The data were collected bimonthly from June 2017 to May 2018 in two different and unconnected aquatic systems in the eastern Amazon region of northern Brazil (Figure 2): estuarine stretch (01°04′17.3″ S, 48°18′36.3″ W) approximately 19 km long, with a mean depth of 6–7 m; river upstream of the Tucuruí Hydroelectric Power Plant (03°48′22.9″ S, 49°44′01.3″ W), with an area of approximately 2,917 km^2^ and maximum depths between 58 and 74 m (Fearnside, 2015). Both sites have a hot, humid tropical climate.

### 2.2 Data collection

At each collection, the abiotic characteristics (temperature, salinity, pH, turbidity, and dissolved‐oxygen concentration) of each site were measured in situ using a Horiba U‐50 multiparameter water quality meter. Precipitation data were obtained from the database of the Brazilian National Meteorological Institute (INMET, 2017). The precipitation regime has four distinct seasonal periods: rainy–dry (June–August), dry (September–November), dry–rainy (December–February), and rainy (March–May).

### 2.3 Capture of specimens

Three sampling points were established at each site for the capture of *M. amazonicum* specimens. At each point, 10 wooden shrimp traps, known locally as “matapis,” baited with grated babaçu (*Orbignya speciosa*) fruit pulp were set out (Simonian, 2006). The traps were set at a depth of 1–2 m for a standard period of 12 hr at both sites. The collected prawns were transported to the laboratory, where they were identified based on an appropriate taxonomic reference (Short, 2004) and sexed (Moraes‐Riodades & Valenti, 2004). The total length (TL) of each specimen was measured (in centimeters), its total mass (TM) was determined (in grams), and the gonads were removed and fixed in Bouin's solution for 24 hr.

### 2.4 Light microscopy

The fixed gonads were processed histologically for embedding in paraffin (Prophet, Mills, Arrington, & Sobin, 1995) to obtain a series of 5‐μm sections, which were hematoxylin–eosin stained and then analyzed and photographed under an Eclipse Ci‐S light microscope fitted with a Nikon S‐Ri1 (Japan) digital camera. The ovarian stages of females were classified as immature, maturing, and mature and reorganized according to the shape, color, and histology of the ovaries (Chaves & Magalhães, 1993; Ferreira, Resende, Lima, Santos, & Rocha, 2012). Male morphotypes were classified at Translucent claw (TC), Cinnamon claw (CC), and Green claw (GC) according to their macroscopic characteristics, color, spination, and germ cell organization in the testis (Silva, Ferreira, Von Ledebur, & Rocha, 2009).

### 2.5 Data analysis

The normality of the data distribution was evaluated using the Shapiro–Wilk test, and the homogeneity of variances was verified by Levene's test. The significance level for all analyses was established as *α* = 0.05.

The Mann–Whitney *U* test was used to detect differences in abiotic factors (pH, salinity, temperature, electrical conductivity, turbidity, and precipitation) between the two environments, estuary and river.

An analysis of covariance (ANCOVA) was used to evaluate the variation in body mass recorded between sites and among periods of the seasonal precipitation cycle, relating the body mass (dependent variable) and the sites and seasonal periods (independent variables) to the total length, dissolved oxygen, temperature, and precipitation as covariables. Analysis was performed after log transformation of data.

Deviations in the sex ratio recorded at each site and during each period of the seasonal precipitation cycle were evaluated using chi‐square (*α* = 0.05) (Zar, 1999). Differences in the mean body length and total mass between sexes, sites, and seasonal periods were tested using two‐way ANOVA in the package *dplyr* (Wickham, François, Henry, & Müller, 2019).

The body mass–length ratio was obtained from the model TM = a*TLb (Huxley, 1924), where TM is the total mass of the specimen, *a* is the coefficient of proportionality, TL is the total length of the specimen, and *b* is the allometric coefficient. In this analysis, a value of *b* equal to 3 corresponds to isometric growth, where the body length of the prawn increases in the same proportion to its mass, *b* < 3 grows faster in length than in weight, and *b* > 3 grows faster in weight than in length.

The mass–length ratios were compared between males and females in the same aquatic system with ANCOVA and between aquatic systems for the same sex. The condition factor (*K*) was calculated by *K* = TM/TL*^b^*, where *b* is the coefficient of allometry determined a priori separately for each sex and site and compared with a one‐way analysis of variance (ANOVA) followed by Tukey's *post hoc* test (Wickham et al., 2019). All analyses were run in the R program version R 3.4.4 (R Core Team, 2018), and both ANCOVA and ANOVA were performed using the package *agricolae* (de Mendiburu, 2019).

## 3 RESULTS

The abiotic factors pH, salinity, temperature, dissolved oxygen, electrical conductivity, turbidity, and precipitation showed significant differences (Mann–Whitney *U*, *p* < .05) between the two environments during the period of this study.

**FIGURE 3:**
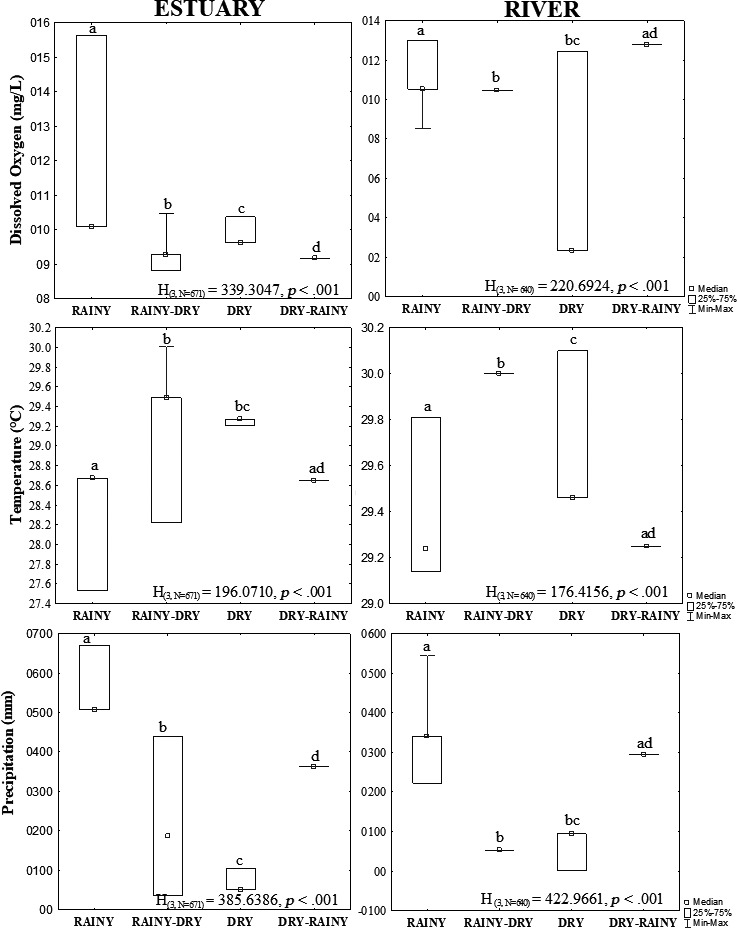
Mean ± standard deviation, and the minimum and maximum values recorded for each environmental variable: dissolved oxygen, water temperature, and precipitation in the estuary and river. Significance level 5%

The locality, seasonal period, and the covariables dissolved oxygen, temperature, and rainfall, all on logarithmic scale, were responsible for the observed variation between the two populations, with significant effects on the body mass of *M. amazonicum* (Table 1). In each environment (estuary and river), the covariates (dissolved oxygen, temperature, and precipitation) responsible for the differences between the mass of the populations showed significant differences during each seasonal period (Kruskal–Wallis, *p* < .001) (Figure 3).

**TABLE 1:** Covariance analysis (ANCOVA) of the relationships between total mass (g) and length (TL), site, period, and abiotic factors for *Macrobrachium amazonicum* in the estuary and river

Relationship	Sum of squares	*df*	Mean square	*F*‐ratio	*p*
Total length	192.482	1	192.482	7,345.556	.000*
Site	1.307	1	1.307	49.882	.000*
Period	2.342	3	0.781	29.795	.000*
Dissolved oxygen	0.123	1	0.123	4.686	.031*
Temperature	0.568	1	0.568	21.673	.000*
Precipitation	1952	1	1.952	74.511	.000*
Site × Period	506	3	0.169	6.437	.000*
Error	33.908	1,294	0.026		

* Statistically significant values.

A total of 1,311 *M. amazonicum* specimens were collected, 671 in the estuary (233 males and 438 females) and 640 in the river (63 males and 577 females). Most specimens in the estuary were collected during the rainy–dry and dry periods, whereas in the river, more specimens were collected during the dry and rainy periods. A female‐biased sex ratio was recorded at both sites during all periods, except the rainy–dry period in the estuary (Table 2).

**TABLE 2:** Number of females (F) and males (M) of *Macrobrachium amazonicum* in the estuary and river, sex ratio, and chi‐square value (*X* ^2^)

Site	Season	F	M	♂:♀	*X^2^*	*p‐value*
Estuary	Rainy	84	35	1:2.4	20.2	<.001
	Rainy–Dry	132	103	1:1.3	3.6	.058
	Dry	145	61	1:2.4	34.3	<.001
	Dry–Rainy	77	34	1:2.3	16.7	<.001
River	Rainy	306	44	1:7.0	196.1	<.001
	Rainy–Dry	30	5	1:6.0	17.9	<.001
	Dry	148	9	1:16.4	123.1	<.001
	Dry–Rainy	93	5	1:18.6	79	<.001

Note

Statistical significance at 0.05 level.

In the estuary, the total length of the females ranged from 4.1 to 12.2 cm and their total mass from 0.4 to 12.4 g; for males, total length ranged from 5.0 to 13.5 cm and total mass from 0.7 to 18.5 g. In the river, the total length of the females was 3.0–7.0 cm and the total mass 0.3–2.7 g, while the males were 3.5–7.0 cm long, with a mass of 0.4–2.8 g (Table 3).

**TABLE 3:** Total length (TL) in centimeters and mass (W) in grams for females (F) and males (M) of *Macrobrachium amazonicum* in the estuary and river, among periods of the year

Site	Period	Sex	TL range	TL mean ± *SD*	*W* range	*W* mean ± *SD*
Estuary	Rainy–Dry	F	4.1‒12.2	8.7 ± 1.3	0.4‒12.4	5.3 ± 2.4
		M	5.0‒13.5	8.8 ± 1.9	0.7‒18.5	5.3 ± 4.1
	Dry	F	5.0‒11.1	7.8 ± 1.2	1.2‒10.0	3.9 ± 1.7
		M	5.5‒11.7	7.4 ± 1.1	1.3‒11.3	3.2 ± 1.8
	Dry–Rainy	F	5.5‒10.6	7.7 ± 1.1	1.1‒8.0	3.8 ± 1.7
		M	5.7‒13.0	8.7 ± 1.9	1.2‒14.5	5.6 ± 3.8
	Rainy	F	6.4‒11.2	8.4 ± 0.9	2.1‒10.0	4.8 ± 1.5
		M	6.2‒13.5	9.0 ± 1.6	1.8‒18.5	5.8 ± 3.6
River	Rainy–Dry	F	4.5‒7.0	5.6 ± 0.7	0.9‒2.7	1.6 ± 0.5
		M	6.0‒6.8	6.3 ± 0.3	1.3‒2.5	1.7 ± 0.5
	Dry	F	3.0‒6.7	4.6 ± 0.7	0.3‒2.2	0.9 ± 0.4
		M	3.5‒7.0	5.0 ± 1.1	0.4‒2.2	0.9 ± 0.6
	Dry–Rainy	F	3.5‒6.5	4.7 ± 0.6	0.4‒1.9	0.8 ± 0.3
		M	4.5‒6.5	5.5 ± 0.8	0.4‒2.1	1.2 ± 0.6
	Rainy	F	4.0‒7.0	5.4 ± 0.5	0.4‒2.6	1.3 ± 0.4
		M	4.5‒7.0	5.7 ± 0.7	0.4‒2.8	1.5 ± 0.6

**FIGURE 4:**
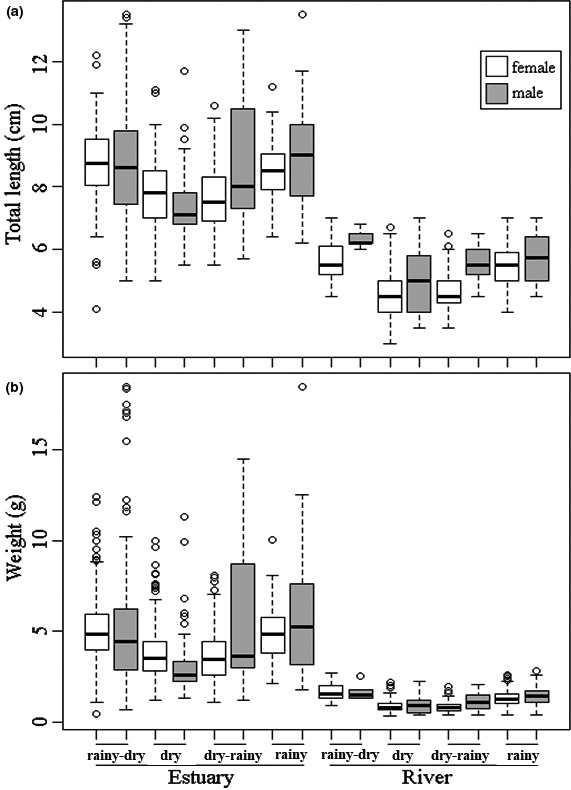
Characteristics of males and females of *Macrobrachium amazonicum* from the estuary and river during hydrological periods, state of Pará, northern Brazil. (a) Total length and (b) total mass

The prawns in the estuary were larger and heavier than those in the river (Figure 4). Mean body length differed significantly between sites, seasons, and sexes (*p* < .001) (Table 4). Body mass differed significantly between sites and seasons (*p* < .001), but showed no significant difference between sexes in either environment (Table 5).

**TABLE 4:** The two‐way ANOVA results for length of the freshwater prawn *Macrobrachium amazonicum*

Factor	*df*	Sum Sq	Mean Sq	*F* value	Pr (>*F*)	Signif.
Site	1	3,147.6	3,147.6	2,748.222	<2E‐016	***
Season	3	261.2	87.1	76.008	<2E‐016	***
Sex	1	10.5	10.5	9.201	0.00247	**
Site:season	3	2.3	0.8	0.662	0.5752	
Site:sex	1	1	1	0.906	0.34133	
Season:sex	3	31.9	10.6	9.284	4.47E‐06	***
Site:season:sex	3	8.9	3	2.577	0.05237	.
Residuals	1,295	1,483.2	1.1			

Note

Signif. Codes: 0 ‘***’ 0.001 ‘**’ 0.01 ‘*’ 0.05 ‘**.**’ 0.1 ‘ ’ 1.

**TABLE 5:** The two‐way ANOVA results for weight of the freshwater prawn *Macrobrachium amazonicum*

Factor	*df*	Sum Sq	Mean Sq	*F* value	Pr (>*F*)	Signif.
Site	1	3,913	3,913	1,133.836	<2E‐016	***
Season	3	326	109	31.477	<2E‐016	***
Sex	1	10	10	2.88	0.08992	.
Site:season	3	52	17	5.037	0.00179	**
Site:sex	1	0	0	0.115	0.7349	
Season:sex	3	96	32	9.269	4.57E‐06	***
Site:season:sex	3	18	6	1.692	0.16695	
Residuals	1,295	4,469	3			

Note

Signif. Codes: 0 ‘***’ 0.001 ‘**’ 0.01 ‘*’ 0.05 ‘**.**’ 0.1 ‘ ’ 1.

No significant difference (ANCOVA, *p* = .48) was found in the body mass/length ratio of the males in the estuary and river, although the females did differ significantly (ANCOVA, *p* < .001) between environments, and all individuals (males and females) combined also differed significantly (Table 6). In all cases, the *b* coefficient was less than 3, which indicates negative allometric growth (Table 6).

**TABLE 6:** Mass–length relationship and condition factor (K) for females (F) and males (M) of *Macrobrachium amazonicum* in the estuary and river

Site	Sex	*a*	95% CL (*a*)	*b*	95% CL (*b*)	*r^2^*	*K*
Estuary	F	0.0122	0.0102–0.0145	2.7818	2.6978–2.8658	.907	1.228
	M	0.0075	0.0063–0.0089	2.9606	2.8773–3.0439	.956	0.754
River	F	0.0207	0.0178–0.0241	2.4219	2.3293–2.5146	.824	2.096
	M	0.0083	0.0051–0.0136	2.910	2.6249–3.1942	.906	0.681

For the estuary, the condition factor (*K*) was 0.754 in males and 1.228 in females, whereas for the river, the *K* value was 0.681 in males and 2.096 in females (Table 6). The *K* values were significantly higher (ANOVA, *p* < .001) in females than in males in both environments.

**FIGURE 5:**
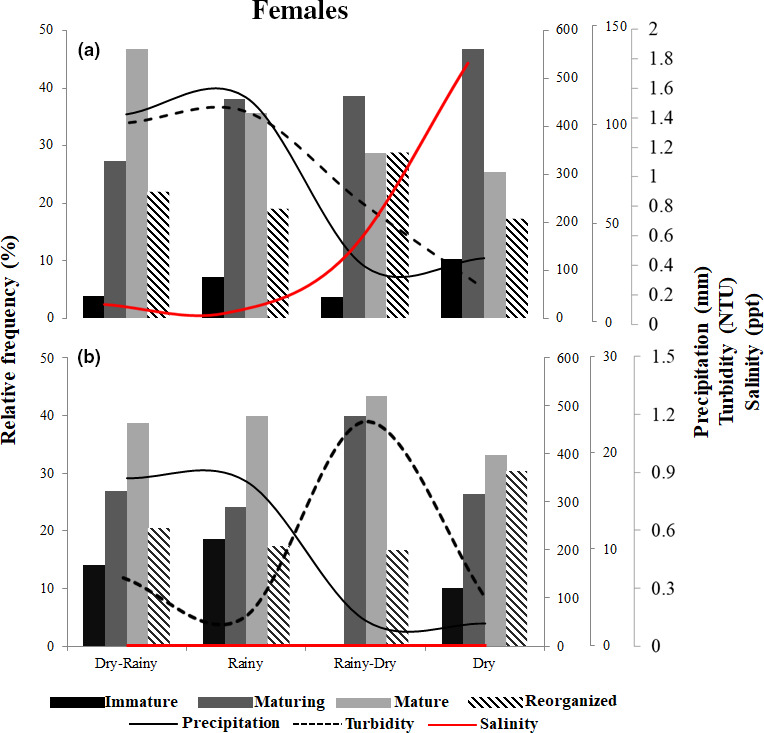
Relationships of the relative frequency of occurrence (%) of maturation stages (immature, maturing, mature, and reorganized) of females of *Macrobrachium amazonicum* to precipitation, turbidity, and salinity in the estuary (a) and river (b)

**FIGURE 6:**
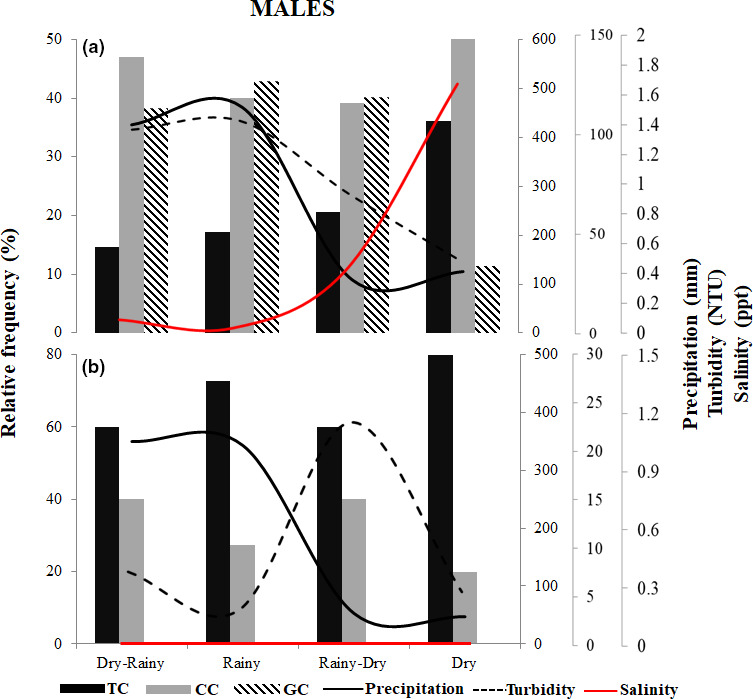
Relationships of the relative frequency of occurrence (%) of morphotypes of males of *Macrobrachium amazonicum* to precipitation, turbidity, and salinity in the estuary (a) and river (b). CC, *Cinnamon claw*; GC: *Green claw*; TC, *Translucent claw*

The relative frequencies of occurrence of the different female maturation stages and the male morphotypes were related to precipitation and turbidity in both environments and also to salinity in the estuary. Mature *M. amazonicum* females were present in all periods of the seasonal cycle in both environments. In the estuary, however, the occurrence of maturing and mature females varied according to the increase in precipitation and turbidity during the dry–rainy and rainy periods and to salinity in the dry period. In the river, maturing and mature females were observed when turbidity was highest, that is, during the rainy–dry period (Figure 5). In males, in the estuary, the TC, CC, and GC morphotypes were observed during all periods, although the dominant GC morphotype was less frequent during the dry period. In the river, the TC and CC morphotypes were observed throughout the year, with a predominance of TC and absence of GC (Figure 6).

## 4 DISCUSSION

In aquatic systems, abiotic factors have a fundamental influence on the life cycle of animal populations, in terms of their ecological, physiological, morphological, genetic, and reproductive characteristics (Augusto, Greene, Laure, & McNamara, 2007; Maciel & Valenti, 2009; Pantaleão, Hirose, & Costa, 2012, 2014; Vergamini et al., 2011). In the two environments analyzed in the present study, one with varying salinity (estuary) and the other with fresh water (river), the above factors were clearly influenced by the seasonal cycle. The data showed that the growth and reproduction of the local populations of *M. amazonicum* in the estuary and river varied considerably among the different seasonal periods. In both environments, precipitation and the other abiotic factors varied significantly. Precipitation is the primary determinant of nutrient concentrations and of the availability of ions and organic material (Maier, 1978).

In the estuary, higher precipitation and salinity rates coincided with higher frequencies of maturing and mature prawns, a pattern that likely affects the reproductive performance of these crustaceans (Costa & Negreiros‐Fransozo, 1998; Meireles et al., 2013). During the rainy season, the high concentrations of nutrients and particulate and dissolved organic matter contributed to an increase in turbidity (de Moura & Nunes, 2016; Tripathi & Pandey, 2014). In the river, in contrast, precipitation and turbidity were inversely proportional, with the reproductive stages of *M. amazonicum* most frequent during periods of higher turbidity. This indicates that in the dammed‐river environment where the water is deeper, the reduction in water volume during the rainy–dry period may result in a concentration of nutrients, which would in turn increase the availability of resources for the prawns. In this case, the availability of nutrients and other local factors may determine reproductive parameters (Pantaleão, Carvalho‐Batista, Teodoro, & Costa, 2018).

Females predominated in the *M. amazonicum* populations in both environments through almost the entire study period. A female‐biased sex ratio is common in crustaceans, given the importance of females for the recruitment process, especially in populations that breed continuously (Ayres‐Santos, Freitas, & Montag, 2018; Castelo‐Branco, Batista, Guerra, Soares, & Peixoto, 2015; Mendes et al., 2017; Mossolin & Bueno, 2002; Sampaio, Silva, Santos, & Sales, 2007). Even so, the males were larger than the females, which may reflect dominance behavior and territoriality, which favor larger individuals (Magalhães, Mossolin, & Mantelatto, 2012; Silva, Frédou, & Filho, 2007). The larger size of the males in comparison with the females probably increases reproductive success and provides advantages during intraspecific competition (Alkalay et al., 2014; Andrade, Fransozo, Bertini, Negreiros‐Fransozo, & López‐Greco, 2015; Gherardi & Micheli, 1989).

The freshwater prawns in the estuary were both longer and heavier than those in the river, with variations in the different environments and seasonal periods. Body mass did not differ significantly between the sexes in either environment. Some authors have found that environmental characteristics may contribute to variation in local populations, such as the influence of coastal dynamics and hydrogeographic aspects of each location, including riparian vegetation, water quality, and predation (de Barros‐Alves et al., 2012; Meireles et al., 2013; Telesh & Khlebovich, 2010). Nevertheless, despite their differences, the estuary and river populations belong to the same monophyletic clade and to a single species (Mishler & Theriot, 2000; Vergamini et al., 2011). Our results indicated a strong influence of the abiotic factors analyzed on the biometric parameters of the prawn.

Deep‐water environments may be characterized by thermal stratification, a reduction in the availability of refuges where marginal vegetation has been lost, a decline in organic matter, low fertility, and an increase in predation (Agostinho et al., 2008; Wang et al., 2013). It seems likely that the freshwater prawns in the river faced limitations of resources that required a larger energy investment in growth.

In both environments, both sexes of freshwater prawns showed negative allometric growth, which means that the freshwater prawn grows faster in length than in weight. Similar findings have been obtained in other studies with *M. amazonicum* (Freire, Marques, & Silva, 2012), and contrast with *M. rosenbergii*, which showed positive allometric growth in culture (Primavera, Parado‐Estepa, & Lebata, 1998) and isometric growth in its natural environment (Kunda et al., 2008). In penaeid prawns, differences between the sexes in the mass–length ratio are common and between prawns in different habitats and seasons (Fontaine & Neal, 1971; Kuris, Ra'anan, Sagi, & Cohen, 2006; Primavera et al., 1998; Von Sperling 2012), which indicates a systematic relationship between habitat and growth patterns in these crustaceans. The negative allometry observed in *M. amazonicum* may be associated with the cycle of gonadal maturation (Freire et al., 2012). Even so, while the freshwater prawns from the estuary were larger and heavier overall, only the females from the river had high K values, which may reflect a larger investment in reproduction.

In the estuary, mature females and all male morphotypes were observed in all seasonal periods, although peaks of maturation and reproduction were recorded during the dry–rainy/rainy periods when precipitation and turbidity increased. Previous studies have shown that the ovarian maturation of freshwater decapods is typically associated with the rainy season (Oh, Ma, Hartnoll, & Suh, 2003). In the river, mature females were also common year‐round but were associated primarily with peaks of turbidity during the rainy–dry period, related to the higher concentrations of suspended organic matter in this environment.

In freshwater decapod crustaceans, ovarian maturation is stimulated by environmental parameters in the natural habitat and is closely associated with rainy periods (Oh et al., 2003). In contrast, variation in the morphotypes of males occupying the same ecological niche has been described in *M. amazonicum* (Moraes‐Riodades & Valenti, 2004; Silva et al., 2009), *M. rosenbergii* (Kuris et al., 2006; Ra'anan & Sagi, 1985), *M. dayanum* (Langer, 2004), and *M. grandimanus* (Wortham & Maurik, 2012). In the present study, while all male morphotypes were recorded in the estuary, GC morphotype males were absent from the river. Individual males may not necessarily pass through all the different phases and may transition from one morphotype to another through either a single molt or a more gradual process (Moraes‐Riodades & Valenti, 2004). It seems likely that the conditions in the river are unfavorable for investment in molting from one morphotype to another, and in this case, the males may remain in the TC morphotype to allow reproductive success in this environment.

Overall, then, the results of this study have shown that different abiotic factors in the estuary and river affect the dynamics of the tolerance and survival of these crustaceans. These conditions may determine population‐level shifts that are fundamental for understanding the life‐history traits of this freshwater prawn. Due to the river damming, there are triggered changes in the freshwater river ecosystem, and this situation played a key role in determining the life‐history characteristics of crustaceans, as, *M. amazonicum*.

## CONFLICT OF INTEREST

I declare that the authors have no financial/personal interest and that the manuscript is for scientific purposes only.

## AUTHOR CONTRIBUTION

**Gicelle M. F. Farias:** Conceptualization (lead); Formal analysis (lead); Investigation (lead); Methodology (lead); Supervision (lead); Visualization (lead); Writing‐original draft (lead); Writing‐review & editing (lead). **Marcelo C. Andrade:** Data curation (supporting); Methodology (supporting); Software (supporting). **Breno R. M. Silva:** Data curation (supporting); Methodology (supporting). **Ingrid S. Palheta:** Data curation (supporting); Methodology (supporting). **Liziane B. Gonçalves:** Data curation (supporting); Formal analysis (supporting); Methodology (supporting). **Rossineide M. Rocha:** Conceptualization (supporting); Resources (supporting); Supervision (supporting); Writing‐original draft (supporting). **Maria A. P. Ferreira:** Conceptualization (equal); Data curation (equal); Formal analysis (equal); Methodology (equal); Resources (equal); Supervision (equal); Writing‐original draft (equal); Writing‐review & editing (equal).

## Data Availability

We declare that all data in this scientific manuscript will be available upon request. Upon acceptance of the article, the data will be archived at https://doi.org/10.5061/dryad.z8w9ghx8f.
